# Effect of 1,25-dihydroxyvitamin D3-glycosides on postpartum health, uterine involution and litter performance of sows in a free farrowing system

**DOI:** 10.1186/s40813-023-00349-3

**Published:** 2023-12-07

**Authors:** Laura Jahn, Alexander Grahofer

**Affiliations:** Clinic for Swine, Department for Clinical Veterinary Medicine, Vetsuisse Faculty, University of Bern, Bremgartenstrasse 109a, 3012 Bern, Switzerland

## Abstract

**Background:**

Vitamin D is essential for the reproductive efficiency in animals. There are indications that 1,25-vitD influenced the farrowing process and thus can decrease postpartum health problems. Therefore, the aim of this study was to investigate the effect of 1,25-vitD on postpartum health of the sow and uterine involution as well as the litter performance.

**Results:**

The rectal body temperature in the 1,25-vitD group was significantly (*p* < 0.05) lower during the first five days after farrowing compared to the negative control group (D1: 38.6 vs. 38.9 °C; D2: 38.5 vs. 38.7 °C; D3: 38.4 vs. 38.7 °C; D4: 38.4 vs. 38.6 °C; D5: 38.5 vs. 38.9 °C). Although there was no difference between the live born piglets after farrowing, a significant higher number of piglets at day 7 and at weaning (Dw) was detected in the 1,25-vitD group compared to the control group (D7: 14.1 ± 0.9 vs. 13.4 ± 1.0, *p* = 0.002; Dw: 14.0 ± 0.9 vs. 13.4 ± 1.0, *p* = 0.02). Furthermore, the litter weight gain was significantly higher in the 1,25-vitD group when compared to the control group (94.3 vs. 86.4 kg; *p* = 0.045), and the weight loss of the sows was significantly lower (52 vs. 59 kg; *p* = 0.03). No differences in other parameter were detected.

**Conclusion:**

This study showed a positive effect of 1,25-vitD on the body temperature, the litter performance and the body condition of the sows during lactation in comparison with the negative control group. However, more studies are needed to describe the mechanism of 1,25-vitD in detail.

## Background

The role of vitamin D to improve reproductive efficiency has been studied in animals and humans in the recent years [1–3]. Until today, different dosages of vitamin D and calcidiol (25(OH)D3) have been tested in gestating sows and growing pigs, leading to increased serum calcidiol. However, only a positive effect on the average daily weight gain for suckling piglets, but not for maternal performance and litter characteristics in sows could be proven [4–7]. Notwithstanding, one clinical study described a reduction in fever and postpartal disorders in sows which received 25(OH)D3 [8] in comparison to the control group. In other animals species and humans more valid research results on vitamin D3 are available. In cows it is described that calcidiol reduces the risk of hypocalcemia, retained placenta and metritis [9]. Furthermore, calcitriol treatment improved Ca and P concentration and had an positive effect on the health condition in overconditioned cows after calving [10]. In women, vitamin D3 and its metabolites were investigated in regards to depression and breastmilk condition after parturition [11].

Reproductive disorders are frequently occurring problems in modern pig production and leading to culling of sows in breeding herds [12–14]. Puerperal disorders, especially the postpartum dysgalactia syndrome (PPDS), have an tremendous economic impact and influence animal health and welfare [15]. Diagnosing this syndrome is challenging, because often clinical signs such as mastitis or an increased amount of vaginal discharge is absent [16–19]. However, an increased body temperature after farrowing is often a reliable indicator for this disease complex [15].

Additionally, puerperal disorders can be associated with a delayed uterine involution and impaired placenta expulsion [20, 21]. Most studies used slaughtered sows to investigate uterine involution with the result of rapidly changes to a pregravid-state [22–24]. However, in recent years ultrasonography examination has been described the most feasible examination method for sows reproductive tract in vivo and has been used to diagnose metritis [20, 25–32]. Previous trials verified transcutaneous ultrasonography as a suitable method to evaluate the uterus and uterine involution during puerperium in sows [20, 31, 32].

There are indications that 1,25-vitD influenced the farrowing process and thus can decrease postpartum health problems. Further knowledge of the influence of 1,25-vitD on the postpartum health and uterine involution in sows is needed. Therefore, the aim of this study was to investigate the effect of 1,25-vitD on postpartum health of the sow and uterine involution the first week after farrowing.

## Results

### General condition

Overall, the general condition of the sows was not disturbed. During the whole study period only five animals showed slightly to moderate signs of disturbed behaviour. Detailed information is presented in Table 1. The univariate analysis resulted no significant difference between the two feeding groups.

**Table 1:** Percentage distribution of the general condition of the two study groups (C = negative control group, received no feed additive and 1,25-vitD = 1,25-vitD group, received a defined amount of 1,25-dihydroxyvitamin D3-glycosides: 1th to 84th gestation day: 26 g 1,25-vitD per day; 85th gestation day until farrowing: 30 g 1,25-vitD per day; during the lactation period: 70 g 1,25-vitD per day) from day 1 to day 5 after farrowing. No significant differences between the two groups were observed

Time of sampling	General condition	C (%)	1,25-vitD (%)	*p*-value
Day 1	Not disturbed	97.6	100.0	0.26
	Slightly disturbed	2.4	0.0	
	Moderate disturbed	0.0	0.0	
Day 2	Not disturbed	100.0	96.0	0.20
	Slightly disturbed	0.0	2.0	
	Moderate disturbed	0.0	2.0	
Day 3	Not disturbed	100.0	100.0	–
	Slightly disturbed	0.0	0.0	
	Moderate disturbed	0.0	0.0	
Day 4	Not disturbed	97.6	98.0	0.89
	Slightly disturbed	2.4	2.0	
	Moderate disturbed	0.0	0.0	
Day 5	Not disturbed	100.0	100.0	–
	Slightly disturbed	0.0	0.0	
	Moderate disturbed	0.0	0.0	

### Feed intake

The feed intake varied between the different days postpartum. Detailed information is presented in Table 2. The univariate analysis resulted no significant difference between the two feeding groups.

**Table 2:** Percentage distribution of the feed intake (Normal = complete feed intake; Reduced = partly feed intake; Inappetence = no feed intake) of the two study groups (C = negative control group, received no feed additive and 1,25-vitD = 1,25-vitD group, received a defined amount of 1,25-dihydroxyvitamin D3-glycosides: 1th to 84th gestation day: 26 g 1,25-vitD per day; 85th gestation day until farrowing: 30 g 1,25-vitD per day; during the lactation period: 70 g 1,25-vitD per day) from day 1 to day 5 after farrowing. No significant difference between the two groups were observed

Time of sampling	Feed intake	C (%)	1,25-vitD (%)	*p*-value
Day 1	Normal	95.1	96.0	0.82
	Reduced	0.0	0.0	
	Inappetence	4.9	4.0	
Day 2	Normal	97.6	98.0	0.89
	Reduced	2.4	2.0	
	Inappetence	0.0	0.0	
Day 3	Normal	97.6	94.0	0.41
	Reduced	2.4	6.0	
	Inappetence	0.0	0.0	
Day 4	Normal	95.1	98.0	0.45
	Reduced	4.9	2.0	
	Inappetence	0.0	0.0	
Day 5	Normal	95.1	100.0	0.12
	Reduced	4.9	0.0	
	Inappetence	0.0	0.0	

### Rectal temperature

**Fig. 1:**
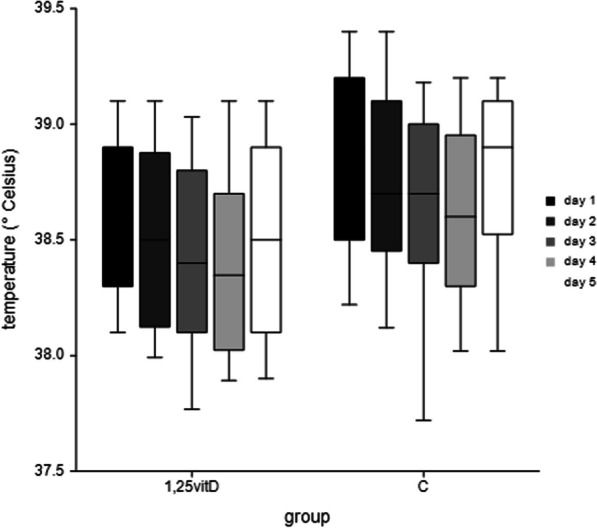
Rectal body temperature (°C) of the two study groups (C = negative control group, received no feed additive and 1,25-vitD = 1,25-vitD group, received a defined amount of 1,25-dihdroxyvitamin D3-glycosides: 1th to 84th gestation day: 26 g 1,25-vitD per day; 85th gestation day until farrowing: 30 g 1,25-vitD per day; during the lactation period: 70 g 1,25-vitD per day) from 1 to day 5 after farrowing. In all days postpartum, the body temperature was significant lower in group 1,25-vitD compared to group C (*p* < 0.05)

On day one postpartum the temperature was in median 38.6 °C (range: 38.0–39.6) for 1,25-vitD and 38.9 °C (range: 38.0–39.6) for C (*p* = 0.02). On day two postpartum the temperature was 38.5 °C (range: 37.3–40.1) for 1,25-vitD and 38.7 °C (range: 36.1–40.2) for C (*p* = 0.02). On day tree postpartum, the temperature was 38.4 °C (range: 37.4–39.5) for 1,25-vitD and 38.7 °C (range: 35.7–39.4) for C (*p* < 0.05). On day four postpartum the temperature was 38.4 °C (range: 36.7–40.1) for 1,25-vitD and 38.6 °C (range: 37.6–40.1) for C (*p* = 0.01). On day five postpartum, the rectal temperature was 38.5 °C (range: 37.4–39.4) for 1,25-vitD and 38.9 °C (range: 37.1–39.6) for C (*p* < 0.01) (Fig. 1). In all days postpartum, the univariate analysis for temperature was significant lower in group 1,25-vitD compared to group C (*p* < 0.05). The further statistical analysis of fever, which was defined with a temperature equal or more than 39.3 °C, showed no differences between the treatment groups.

### Vaginal discharge

The vaginal discharge varied between the two feeding groups and showed only a significant difference in colour on day four postpartum (*p* < 0.05). On this day, white-yellowish vaginal discharge was detected in 60.7% and transparent vaginal discharge in 39.3% of the sows with vaginal discharge in the 1,25-vitD group. Whereas 39.0% transparent and white-yellowish vaginal discharge and 22.2% reddish vaginal discharge was detected in the negative control group. The prevalence of post-partum vaginal discharge overall sowsand in the different groups is presented in Table 3. Further details of the distribution of the colour of vaginal discharge on the different sampling days are presented in Table 4.

**Table 3:** Percentage distribution of the quantity of the vaginal discharge (No = no signs of vaginal discharge, Slight = slight vaginal discharge and Severe = severe vaginal discharge, tail and perineal region contaminated with vaginal discharge)of the two study groups (C = negative control group, received no feed additive and 1,25-vitD = 1,25-vitD group, received a defined amount of 1,25-dihydroxyvitamin D3-glycosides: 1th to 84th gestation day: 26 g 1,25-vitD per day; 85th gestation day until farrowing: 30 g 1,25-vitD per day; during the lactation period: 70 g 1,25-vitD per day) from day 1 to day 5 after farrowing. No significant differences between the two groups were observed

Time of sampling	Vaginal discharge amount	C (%)	1,25-vitD (%)	*p*-value
Day 1	No	74.4	70.2	0.81
	Slight	15.4	14.9	
	Serve	10.3	14.9	
Day 2	No	31.7	40.4	0.18
	Slight	19.5	29.8	
	Serve	48.8	29.8	
Day 3	No	36.6	45.8	0.47
	Slight	34.1	35.4	
	Serve	29.3	18.8	
Day 4	No	56.1	42.9	0.43
	Slight	29.3	40.8	
	Serve	14.6	16.3	
Day 5	No	46.3	53.1	0.55
	Slight	41.5	30.6	
	Serve	12.2	16.3

**Table 4:** Percentage distribution of the colour of the vaginal discharge of the two study groups (C = negative control group, received no feed additive and 1,25-vitD = 1,25-vitD group, received a defined amount of 1,25-dihydroxyvitamin D3-glycosides: 1th to 84th gestation day: 26 g 1,25-vitD per day; 85th gestation day until farrowing: 30 g 1,25-vitD per day; during the lactation period: 70 g 1,25-vitD per day) from day 1 to day 5 after farrowing. Asterisk and bold represent significant differences between the two study groups (*p* < 0.05)

Time of sampling	Vaginal discharge colour	C (%)	1,25-vitD (%)	*p*-value
Day 1	Tansparent	10.0	7.1	0.12
	White–yellow	50.0	85.7	
	Red	40.0	7.1	
Day 2	Transparent	3.6	21.4	0.05
	White–yellow	82.1	75.0	
	Red	14.3	3.6	
Day 3	Transparent	19.2	26.9	0.75
	White–yellow	69.2	65.4	
	Red	11.5	7.7	
Day 4	Transparent	38.9	39.3	0.01*
	White–yellow	38.9	60.7	
	Red	22.2	0.0	
Day 5	Transparent	27.3	41.7	0.37
	White–yellow	45.5	45.8	
	Red	27.3	12.5	

### Mammary gland

There were only single animals with signs of inflammation (redness or heat) of the mammary gland. The first day postpartum redness was present in four sows (one in the 1,25-vitD group and three in the negative control group). In addition, redness could be detected in two sows of the negative control group on the second, third and fifth day postpartum and in one sow on the fourth day postpartum. An increased mammary gland temperature could be detected on day one (one sow of the control group), day three (one sow of the control group) and day five (one sow of the control group and one sow of the 1,25-vitD group). No sows showed signs of pain in mammary gland palpation. The univariate analysis resulted no significant difference between the feeding groups.

### Treatment incidence

Overall, the treatment incidence was 6.4% during the study period. The decision of treatment was made by the farmer. Details of the treatment incidence are presented in Table 5. The univariate analysis resulted no significant difference between the two feeding groups.

**Table 5:** Percentage distribution of different treatments (No = no treatment; Analgesic = Metamizole (35 mg/kg body weight) or Meloxicam (0.4 mg/kg body weight); Antibiotic = Sulfadioxin (12.5 mg/kg body weight) Trimethoprim (2.5 mg/kg body weight) of the two study groups (C = negative control group, received no feed additive and 1,25-vitD = 1,25-vitD group, received a defined amount of 1,25-dihydroxyvitamin D3-glycosides: 1th to 84th gestation day: 26 g 1,25-vitD per day; 85th gestation day until farrowing: 30 g 1,25-vitD per day; during the lactation period: 70 g 1,25-vitD per day) from day 1 to day 5 after farrowing. No significant differences between the two groups were observed

Time of sampling	Treatment	C (%)	1,25-vitD (%)	*p*-value
Day 1	No	78.1	78.0	0.99
	Analgesic	19.5	20.0	
	Analgesic + Antibiotic	2.4	2.0	
Day 2	No	90.2	98.0	0.25
	Analgesic	7.3	2.0	
	Analgesic + Antibiotic	2.4	0.0	
Day 3	No	100.0	98.0	0.36
	Analgesic	0.0	2.0	
	Analgesic + Antibiotic	0.0	0.0	
Day 4	No	97.6	100.0	0.27
	Analgesic	2.4	0.0	
	Analgesic + Antibiotic	0.0	0.0	
Day 5	No	100.0	96.0	0.20
	Analgesic	0.0	4.0	
	Analgesic + Antibiotic	0.0	0.0	

### Uterine horn diameter

Uterine diameter of sows in the 1,25-vitD and control group decreased between days 3 and 24 postpartum from 26.5 to 13.5 mm and 24.55 to 13.5 mm. Further details are presented in Table 6. The uterine horn diameter showed no significant difference between 1,25-vitD and C.

**Table 6:** Uterine diameter in sows day 3, 5, 7 and once between day 23 and 25 after farrowing of the two study groups C = negative control group, received no feed additive and 1,25-vitD = 1,25-vitD group, received a defined amount of 1,25-dihydroxyvitamin D3-glycosides: 1th to 84th gestation day: 26 g 1,25-vitD per day; 85th gestation day until farrowing: 30 g 1,25-vitD per day; during the lactation period: 70 g 1,25-vitD per day) No significant differences between the two groups were observed

Time of sampling	C (mm) Mean ± SD	1,25-vitD (mm) Mean ± SD	*p*-value
Sono d3	25.49 ± 4.2	26.73 ± 4.5	0.17
Sono d5	21.79 ± 3.6	22.26 ± 3.3	0.50
Sono d7	18.63 ± 2.6	19.48 ± 2.7	0.12
Sono d24	13.75 ± 1.8	13.80 ± 2.2	0.98

### Litter performance

Although there was no difference between the live born piglets after farrowing, a significant difference between the number of piglets at day 7 and at weaning was detected (D7: 14.1 ± 0.9 vs. 13.4 ± 1.0, *p* = 0.002; Dw: 14.0 ± 0.9 vs. 13.4 ± 1.0, *p* = 0.02). Furthermore, the litter weight gain was significantly higher in the 1,25-vitD in comparison to the control group (94.3 vs. 86.4 kg; *p* = 0.045). Further details are presented in Table 7.

**Table 7:** Sow traits and litter characteristics for the two study groups (C = negative control group, received no feed additive and 1,25-vitD = 1,25-vitD group, received a defined amount of 1,25-dihydroxyvitamin D3-glycosides: 1th to 84th gestation day: 26 g 1,25-vitD per day; 85th gestation day until farrowing: 30 g 1,25-vitD per day; during the lactation period: 70 g 1,25-vitD per day). Asterisk and bold represent significant differences between the two study groups (*p* < 0.05)

Parameters	C (n) Median (Min;Max)	1,25-vitD (n) Median (Min;Max)	*p*-value
Live born piglets after farrowing (n)	0.0 (4.0; 22.0)	15.0 (5.0; 24.0)	0.68
Number piglets day 7 (n)	14.0(11.0; 15.0)	14.0 (12.0; 15.0)	0.002*
Number piglets at weaning (n)	14.0 (11.0; 15.0)	14.0 (11.0 15.0)	0.02*
Litter weight at farrowing (kg)	22.1 ± 5.0	21.1 ± 4.5	0.30
Litter weight at weaning (kg)	109.0 (60.5; 139.0)	117.0 (60.0; 141.0)	0.06
Litter weight gain (farrowing to weaning) (kg)	86.4 (42.3; 123.1)	94.3 (33.5; 122.3)	0.045*
Sow body weight before farrowing (kg)	265.0 ± 32.0	265.0 ± 35.0	0.97
Sow body weight after weaning (kg)	207.0 (152.0; 265.0)	212.0 (168.0; 272.0)	0.49
Difference in sow weight loss (kg)	59.0 (0.0; 109.0)	52.0 (0.0; 83.0)	0.03*

### Sow body weight

At the time of moving to the farrowing unit, the sows mean body weight was 264 ± 34 kg, for group 1,25-vitD it was 264 ± 35 kg and for negative control group it was 265 ± 32 kg. After weaning there was no normal distribution of body weight, the median was 209 kg (range: 152–272), for group 1,25-vit D it was 212 kg (168–272) and for negative control group it was 207 kg (152–265). The difference between the body weight at arriving and at leaving the farrowing unit was in median 52 kg (0–83) for group 1,25-vitD and 59 kg (0–109) for negative control group.

With Wilcoxon Rank-Sum Test there was no difference between 1,25-vitD and negative control group in the body weight of sows within moving to the farrowing unit (*p* = 0.97) and after weaning (*p* = 0.49). However, the difference between the body weight at farrowing and at weaning became evident between 1,25-vitD and negative control group with 52 vs. 59 kg; *p* = 0.03 (Table 7).

## Discussion

Hence, vitamin D3 in the basal diet is not bioactive, the functional form of vitamin D3 1,25-vitD on top of the usual vitamin D supplementation was used to improve its availability and the performance of sows and their litters. Therefore, this study evaluated the effect of 1,25-vitD supplementation on the postpartum health and uterine involution of sows in a free farrowing system.

To enable a reliable investigation of the effect of a feed additive on postpartum health a randomized parallel design study was chosen. To reduce the performance and attrition bias, three investigators conducted the postpartum health sampling after thorough initial training and using a detailed evaluation form. The uterine involution was conducted by the same investigator every time to minimize the performance bias. Even though 91 sows were included in this study, the sample size per group was relatively small and therefore, additionally stratification on i.e., parity was not possible. Additionally, there are some missing data because of aggressive behaviour of the sows wherefore examination was not possible on every single day postpartum. Another limitation was that only one specific population (one Swiss sow pool system) was examined. Therefore, the housing and management conditions for all sows were identical, allowing a valid comparison of the treatment groups, i.e., the effect of a plant-based source of 1,25-vitD. Even tough, free farrowing sows were used in this study, the postpartum health and uterine involution can be extrapolated to larger populations in other countries, because the treatment effect should not be affected by the housing system.

Puerperal diseases, especially PPDS have a major impact on the further reproductive performance and uterine health in sows and can lead to tremendous economic losses. It is evident that fever is one of the first clinical signs for PPDS [15], but to confirm the diagnosis further indicators such as a reduced general health condition, anorexia, mastitis and/or vaginal discharge can be observed [17, 18]. Therefore, in this study those parameters were assessed to evaluate the health status of the sow and describe the influence of 1,25-vitD on the puerperium. Physiologically, the farrowing procedure initiates an inflammatory process evidenced by a rise of interleukins, an increase of IL-6 and tumour necrosis factor alpha in the blood, which cause a rise in body temperature [33]. Only a significant lower rectal temperature on the five sampling days after farrowing in group 1,25-vitD in comparison with the control group was detected. These findings are in line with a recent study reporting a lower prevalence of fever and postpartum disorders in sows receiving a diet including 50 μg/kg 25(OH)D3 in comparison with sow receiving the standard diet [8]. As vitamin D is a potent immunomodulator [34] it can be hypothesised that added 1,25-vitD might have suppressed pro-inflammatory cytokine and thereby reduced the body temperature in sows. In addition, it is known that a vitamin D deficiency in women is associated with postpartum disorders, such as endometritis [35] or bacterial vaginosis [36]. Furthermore, it is described that the vitamin D blood level in cows or a treatment after calving with a combination of vitamins (AD3E) in buffalos positively influenced the vaginal mucus discharge and the uterine health in the postpartum period [37, 38]. However, in this study no significant differences of the vaginal discharge parameters between the two groups could be detected. Therefore, it can be concluded that 1,25-vitD might have a positive effect on the body temperature in free farrowing sows after farrowing. However, due to a low prevalence of PPDS in the study population, the effect of 1,25-vitD on this disease complex and other postpartum disorders could not be evaluated and thereby warrants further studies.

Although, no significant differences between the two feeding groups could be detected in the general and uterine health of the sows, the number of piglets on day 7 after farrowing and at the day of weaning was significant higher in the 1,25-vitD group. Therefore, the piglet losses during the lactation period were significantly reduced by the feed additive. This is in line with two studies, were sows fed with 25(OH)D3, a more readily bioavailable and efficiently absorbed vitamin D3 metabolite than vitamin D3, revealing a lower mortality rate of piglets in comparison with vitamin D3 [39–41]. The detailed causes for this effect remained unknown. However, it can be speculated that through the significant lower body temperature in the 1,25-vitD group, sows might have been more alert for piglet distress, had a lower frequency of un-rewarded sucking attempts and showed more maternal behaviour, which might lead to less piglet mortality in the lactational period. Furthermore, the increased daily weight gain of the piglets in the 1,25-vitD group might have led to this effect, due to a higher vitality of the piglets.

A trend of an increased litter weight at weaning and a significant difference in the daily weight gain of the piglets from 1,25-vitD in comparison with the negative control group was detected. This is in line with current literature, where 25(OH)D3 showed the same significant effects as 1,25-vitD in comparison with vitamin D3 [39, 41]. It might be that similarly as for 25(OH)D3 that 1,25-vitD influences the growth promoting hormones, especially the insulin like growth factor. Therefore, it might be that the increased daily weight gain, is caused by an increase in the insulin like growth factor [34, 38, 42].

Interestingly, the body weight loss was significant less reduced in the 1,25-vitD group. However, this is in contrast with several studies with 25(OH)D3, another vitamin D3 metabolite and therefore further studies are needed for valid results.

Puerperal diseases are often associated with a delay in uterine involution [20, 21, 28]. It is known that 25(OH)D3 can reduce the risk of metritis in cows [9] and decreases postpartum problems in sows [8]. In this trial no significant difference between the two treatment groups in the uterine horn diameter could be detected by ultrasonography examination. This can be due to the normal feeding ratio containing 1000 UI Vitamin D3/kg or the free farrowing system [32]. Another hypothesis is that 1,25-vitD has no influence on the uterine involution in sows. Nevertheless, already published uterine diameter values [21, 32] could be confirmed in this study and are useful for further studies.

In conclusion, this study showed a positive effect of 1,25-vitD on the body temperature, litter performance and the weight loss of the sows during lactation in comparison with the negative control group (1000 IU Vitamin D3/kg). However, more studies are needed to describe the mechanism of 1,25vitD in detail and prove the effect in sows with PPDS.

## Methods

### Experimental design

In this study a randomized parallel study design was conducted in a Swiss sow pool system to investigate the effect of a plant-based source of 1,25-vitD on postpartum health, uterine involution and litter performance of 100 sows. Sows with a poor general condition or sever lameness before farrowing were excluded from the study population. The responsible Veterinary Office of Solothurn, Switzerland approved the study protocol (licence no. SO 02/2020; No. 33057).

All sows (Swiss Large White × Landrace) were housed under similar housing conditions in a Swiss sow pool system. The treatment group (1,25-vitD) received the same commercial feed as the negative control group (C) (1000 IU Vitamin D3/kg) with a defined amount of feed additive which contained standardized level of 1,25(OH)2D3-gly (1,25-vitD; Herbonis Panbonis^®^). Between the first to 84th gestation day the sows received 26 g 1,25-vitD per day (260 mg/day/sow), from the 85th gestation day until farrowing the sows received 30 g 1,25-vitD per day (300 mg/day/sow) and during the lactation period, the sows received 70 g 1,25-vitD per day (700 mg/day/sow). These dosages correspond to the ones in the registration of the product. Since the effect on the blood level of vitamin D with the 1,25-dihydroxyvitamin D3-glycosides of Herbonis have been already proven by the feeding company during the registration process and to reduce stress in the peripartal period and don’t influence maternal behaviour, blood sampling of the sows in this study was not conducted.

The pre-farrowing and farrowing data have been published previously [43]. In this study, the postpartum health status of the sows was evaluated once a day from the first until the fifth day after farrowing. The following parameter were assessed: general condition, feed intake, rectal temperature, vaginal discharge and health of mammary gland of sows once a day. The general condition was evaluated using the following scoring system from 0 to 3 (zero: good general condition (no disturbance in general condition); one: slightly disturbed general condition (somnolent behaviour: frequent and protracted sitting, with drooping of the head and eyes half closed.; two: moderately disturbed general condition (stupor behaviour: excessively deep state of unresponsiveness, responsive to external or internal stimuli); three highly disturbed general condition (coma behaviour: not responsive to external or internal stimuli). Feed intake was evaluated by visual examination one hour after feeding on a trichotomous scale (normal feed intake: complete feed intake; reduced feed intake: partly feed intake; inappetence: no feed intake). The quantity of the vaginal discharge was categorized into score 0 (no signs of vaginal discharge), score 1 (slight vaginal discharge) and score 2 (severe vaginal discharge, tail and perineal region contaminated with vaginal discharge). The colour of the vaginal discharge was classified into clear, whitish/yellowish and reddish [44]. The following parameters of the mammary gland were assessed on a dichotomous scale (Yes/No): redness, swelling, tenderness, and heat of each mammary complex. Retrospectively, the rectal body temperature was grouped into greater or equal 39.3 °C (fever) and less than 39.3 °C (no fever).

In addition, transabdominal ultrasonography was used to examine the puerperal uterus of each sow at day three, five and seven and again once between day 23 and day 25 after farrowing. Sows were examined according the previously published method [32]. The ultrasonic equipment used was a MyLab™OneVet Esaote Spa^©^ in combination with the abdominal convex probe SC3421 (frequency 1–8 MHz). The following settings were used to evaluate the uterine involution: 6.6 MHz, 12 cm penetration depth with a dynamic range at 7.

Retrospectively, the ultrasonographic images as well as videos were analysed using the imaging software MyLab_Desk 9.0. For the diameter measurement, cross-sections of round shape were identified and measured in two dimensions to use the mean diameter. The results obtained from the measurement of three cross-sections/sow/day averaged the mean diameter per cross-section/sow/day.

Furthermore, the treatment incidence was assessed using a trichotomous scale (No treatment, analgesic treatment and a combination of analgesic and antibiotic treatment).

In addition, litter performance and weight loss of the sows during the lactational period were evaluated. The number of live born piglets at farrowing, at day 7 and at weaning was assessed. Cross fostering of piglets within the assigned group was conducted by the farmer, based on his experience, and working routine between 12 and 36 h after farrowing. The total litter weight (live born piglets, kg) at farrowing and weaning were determined by using a platform scale (Professional 3700, Soehnle Industrial Solutions GmbH, Backnang, Germany). The sows were weighted before entering the farrowing unit and at the day of weaning. Retrospectively, the weight gain of the litters as well as the weight loss of the sows was calculated.

### Statistical analyses

Data were collected digitally using a database program (Microsoft Access 2016) and transferred into a spreadsheet program (Microsoft Excel 2016). Statistical processing of all data conducted using NCSS 2020 Data [NCSS 2020 Statistical Software (2020). NCSS, LLC. Kaysville, Utah, USA, ncss.com/software/ncss].

All continuous data were tested for normality using the Shapiro–Wilk normality test. If there were not normally distributed, they were tested again with logarithmic parameters. Equal variance t-test was used to compare normally distributed data. Not normally distributed data were analysed with Wilcoxon Rank–Sum Test.

All nominal or binary data was tested for independence with Pearson’s Chi-Square test or Likelihood Ratio if there were less than five values in one group.

For further use, treatment was grouped into no treatment, analgesics or analgesic and antibiotics. All tests were determined to be statistically significant with a confidence interval of 95% and *p*-values less than 0.05.

## Data Availability

The dataset for this study is available from the corresponding author on reasonable request.
